# Supplementary material for: Single gene analysis in yeast suggests nonequilibrium regulatory dynamics for transcription
Source: Nat Commun. 2024 Jul 23;15:6226. doi: 10.1038/s41467-024-50419-5 (PMC11266658; doi:10.1038/s41467-024-50419-5)
Supplement: Supplementary file 1 — Supplementary Information [file 41467_2024_50419_MOESM1_ESM.pdf]

Single Gene Analysis in Yeast Suggests Nonequilibrium Regulatory Dynamics for Transcription

SUPPLEMENTAL INFORMATION

Here we provide supplemental data (Section V) and lay out the mathematical arguments of our analysis (Sections I - IV). We show that the probability density function f_A of H_A, the dwell time of the stochastic process in mesostate A, is given by the sum of weighted exponential decay functions, provided that specific mathematical conditions are fulfilled (Section I). We show that all these conditions hold for any process, reversible or not, on graphs with three nodes (Section II), and for reversible processes on any graph (Section III). We prove that f_A is monotonic for reversible processes (Section III, Theorem 4). In section IV we show that any coarse-grained process provides a lower bound on the true entropy production (Theorem 5).

I. Dwell-time densities (Theorem 1), pp. 1-6.

II. Three-state graphs (Theorems 2 and 3), pp. 6-10.

III. Tu's theorem (Theorem 4), pp. 10-14.

IV. Entropy production of coarse-grained processes (Theorem 5), pp. 14-16.

V. Extended Data figures, pp 16-19.

VI. Citations, pp. 19-20.

I. DWELL TIME DENSITIES

Let $Y = \{Y_t: \Omega \longrightarrow S | t \in \mathbb{R}_{\leq 0}\}$ be a random process, i.e., a family of random variables Y_t that map Ω, the "sample space," onto S, the "state space," which we assume is enumerable and finite, $S = \{1, \ldots, n\}$. We partition S into two disjoint subsets, A and B (ON and OFF), i.e., $S = A \cup B$ and $A \cap B = \emptyset$. We refer to elements of S as "microstates", and to A and B as "mesostates." The lifetime of A (the dwell time of the process in A) is a continuous random variable, H_A ([1]).

Let $m < n$ the number of microstates that pertain to A ("A states"). The number of microstates that pertain to mesostate B ("B states") is therefore $n - m$. We assume that Y is a time-homogeneous Markov process, i.e., the probability current from state i to j is given by

$$v_{ji}(t) = w_{ji} p_i(t) \ ^1, \qquad [1]$$

([1]) We use H_A (the "holding time" in A) instead of T (the lifetime, *cf.* main text) to avoid confusion with the transpose, T (*cf.* Footnote 2).

where $p_i(t)$ is the probability that the process resides in state i at time t and w_{ji} is a rate constant. The *column* vector $\boldsymbol{p}(t) = (p_1(t),\dots,p_n(t))^T$ is called the probability mass function at time t ([2]). Provided the graph of the process is "strongly connected" — i.e., every state may be reached from any other state by a string of transitions — the process approaches a uniquely defined distribution (independently of the initial distribution) [2]. This limit distribution is called the "stationary distribution," $\boldsymbol{p}$ of Y. The process, then, is in "steady state," i.e., $dp_i(t)/dt = 0$ for all $i \in S$.

The "generator" of the process is the square matrix of rate constants:

$$\mathrm{W} = \begin{pmatrix} w_{11} & \cdots & w_{1n} \\ \vdots & \ddots & \vdots \\ w_{n1} & \cdots & w_{nn} \end{pmatrix},$$

where

$$w_{jj} \equiv -\sum_{k \neq j} w_{jk} \; ; \qquad [2]$$

in words, the diagonal element w_{jj} is the negative sum of the remaining elements in column j. The generator determines the time-evolution of probability mass across the state space [3], according to

$$\frac{d\boldsymbol{p}(t)}{dt} = \mathrm{W}\boldsymbol{p}(t)\,,$$

the "master equation" of the process [4].

We enumerate the elements of S such that states $1,\dots,m$ are A states and states $m + 1,\dots,n$ are B states. Correspondingly, we partition the generator into the following sub-matrices:

$$\mathrm{W}_{AA} = (w_{ji}) \text{ where } i,j \in A;\;\; \mathrm{W}_{AB} = (w_{ji}) \text{ where } j \in A, i \in B;\; etc.$$

The generator, then, is

$$\mathrm{W} = \begin{pmatrix} \mathrm{W}_{AA} & \mathrm{W}_{AB} \\ \mathrm{W}_{BA} & \mathrm{W}_{BB} \end{pmatrix}.$$

(Note that the diagonal elements of W are the diagonal elements of W_{AA} and W_{BB}.) Other matrices, including vectors, are partitioned accordingly — e.g., $\boldsymbol{p} = (\boldsymbol{p}_A, \boldsymbol{p}_B)^T$. The vectors $\boldsymbol{u}_A$ and $\boldsymbol{u}_B$ designate the *row* vectors $(1,\dots,1)$ of length m and $n - m$, respectively.

For the probability that the process leaves A no earlier than t but no later than $t + h$, given that the process started in $i \in A$ at $t = 0$, we write

$$P(t \leq H_A < t + h | Y_0 = i \in A)\,.$$

([2]) The superscript T indicates the transpose, the exchange of columns with corresponding rows.

To find this probability, which is given by the integral over the density function of H_A between t and $t + h$, we follow Colquhoun and Hawkes and consider the *modified* process in which all B states are absorbing [5]. Thus, $j \in B$ is reached from $i \in A$ by crossing the boundary between A and B no more than once (*i.e.*, $\mathrm{W}_{AB} = \mathbf{0}$), and probability mass does not disperse between B states (*i.e.*, $\mathrm{W}_{BB} = \mathbf{0}$). ([3])

For the *modified* process, let

$$F_{ji}(t) = P(Y_t = j|Y_0 = i)$$

be the probability that the process is in state j at time t, given that the process was in state i at time 0 for $i, j \in S$. Furthermore, let $F = (F_{ji})$ be the matrix of all "transition functions" F_{ji}.

In the following, let $j \in B$ and $i \in A$. For time homogenous processes,

$$P(Y_{t+h} = j|Y_h = i) = P(Y_t = j|Y_0 = i)$$

for all $h \geq 0$, and we may thus write

$$F_{ji}(t+h) = \sum_{k \in S} F_{jk}(h) F_{ki}(t) \,. \qquad [3]$$

(Eq. [3] is called the "Chapman-Kolmogorov equation [6].") Since B states are absorbing, $F_{jk}(h) = 0$ for all $k \in B$ with $k \neq j \in B$, whereas $F_{jk}(h) = 1$ for $k = j$. Hence,

$$F_{ji}(t+h) = \sum_{k \in A} F_{jk}(h) F_{ki}(t) + F_{ji}(t) \,. \qquad [4]$$

Note that

$$F_{ji}(t+h) - F_{ji}(t) = P(t \leq H_A < t+h, Y_{t+h} = j|Y_0 = i \in A\,) \qquad [5]$$

is the probability that the transition from A to $j \in B$ occured within the interval $(t, t+h]$, given the process started in state $i \in A$.

Since $F_{jk}(0) = 0$ for all $k \in A, j \in B$, we may rewrite Eq. [4]:

$$F_{ji}(t+h) - F_{ji}(t) = \sum_{k \in A} \left(F_{jk}(h) - F_{jk}(0)\right) F_{ki}(t) \,. \qquad [6]$$

Division by h and taking the limit for $h \to 0$ gives

$$\frac{dF_{ji}(t)}{dt} = \sum_{k \in A} w_{jk} F_{ki}(t) \,, \qquad [7]$$

since

([3]) Here and in the following, **0** is the zero matrix; its dimensions will be clear from the context; for instance, **0** may be a column or row vector.

$$\frac{F_{jk}(h) - F_{jk}(0)}{h} \longrightarrow w_{jk}$$

for $h \to 0$; i.e., the rate constants are the time-derivates of the transition functions at time 0 [3].

Summing Eq. [5] over all $j \in B$, we obtain

$$P(t \le H_A < t + h | Y_0 = i \in A) = \sum_{j \in B} \left(F_{ji}(t + h) - F_{ji}(t) \right). \qquad [8]$$

The conditional density function for H_A, given the process started in $i \in A$, is now obtained by dividing Eq. [8] by h and taking the limit for $h \to 0$:

$$f_i(t) = \sum_{j \in B} \frac{dF_{ji}(t)}{dt} = \sum_{j \in B} \sum_{k \in A} w_{jk} F_{ki}(t) \qquad [9]$$

for all $i \in A$, where the second equality follows with Eq. [7].

Let $\boldsymbol{f}_A(t)$ be the *row* vector whose i^{th} component is $f_i(t)$, where $i \in A$. Thus, we may write equations [9] in matrix form:

$$\boldsymbol{f}_A(t) = \boldsymbol{u}_B \mathrm{W}_{BA} F_{AA}(t) . \qquad [10]$$

The transition matrix $F(t)$ is determined by the differential equation:

$$\frac{dF(t)}{dt} = \begin{pmatrix} \mathrm{W}_{AA} & \mathbf{0} \\ \mathrm{W}_{BA} & \mathbf{0} \end{pmatrix} \begin{pmatrix} F_{AA}(t) & F_{AB}(t) \\ F_{BA}(t) & F_{BB}(t) \end{pmatrix} = \begin{pmatrix} \mathrm{W}_{AA} F_{AA}(t) & \mathbf{0} \\ \mathrm{W}_{BA} F_{BA}(t) & \mathbf{0} \end{pmatrix} \text{ (4)} \qquad [11]$$

which implies

$$\frac{dF_{AA}(t)}{dt} = \mathrm{W}_{AA} F_{AA}(t) . \qquad [12]$$

The solution of the last equation is [6]

$$F_{AA}(t) = e^{t \mathrm{W}_{AA}} = \sum_{k=0}^{\infty} \frac{(t \mathrm{W}_{AA})^k}{k!} . \qquad [13]$$

The process begins its sojourn in A only in states that are accessible from B. The probability that the process begins in $i \in A$, given that it begins in A, is the probability that the unmodified process, when it leaves B, enters i. This probability is equal to the probability current (i.e., probability mass per unit times) from B into $i \in A$,

$$v_i(t) = \sum_{j \in B} w_{ij} \, p_j(t) ,$$

(4) Note that for the modified process, $F_{AB}(t) = \mathbf{0}, F_{BB}(t) = \mathbf{0}$ for all t.

divided by the total probability current from B to A,

$$\boldsymbol{u}_A \boldsymbol{v}(t) = \sum_{i \in A} v_i(t) \,,$$

where $\boldsymbol{v}(t) = (v_1(t), \dots, v_m(t))^T$. Thus, the probability that the process begins in state $i \in A$, given that it begins in A, is

$$r_i(t) = v_i(t) / \boldsymbol{u}_A \boldsymbol{v}(t) \,.$$

With $\boldsymbol{r}(t) = (r_i(t), \dots, r_m(t))^T$ it follows that

$$\boldsymbol{r}(t) = \frac{\boldsymbol{v}(t)}{\boldsymbol{u}_A \boldsymbol{v}(t)} = \frac{\mathrm{W}_{AB} \boldsymbol{p}_B(t)}{\boldsymbol{u}_A \mathrm{W}_{AB} \boldsymbol{p}_B(t)} \,. \qquad [14]$$

The density function of H_A, then, is given by

$$f_A(t) = \boldsymbol{f}_A(t) \boldsymbol{r}(t) \,. \qquad [15]$$

Insertion of Eqs. [10], [13] and [14] into Eq. [15] gives

$$f_A(t) = \boldsymbol{u}_B \mathrm{W}_{BA} e^{t \mathrm{W}_{AA}} \frac{\mathrm{W}_{AB} \boldsymbol{p}_B(t)}{\boldsymbol{u}_A \mathrm{W}_{AB} \boldsymbol{p}_B(t)} \,. \qquad [16]$$

To calculate $e^{t\mathrm{W}_{AA}}$, and thus $f_A(t)$, we now assume that W_{AA} is diagonalizable — i.e., W_{AA} has m linearly independent eigenvectors. ([5]) According to the spectral decomposition theorem of linear algebra, W_{AA} may then be written as a linear combination,

$$\mathrm{W}_{AA} = \sum_{i \in A} \lambda_i \mathrm{A}_i \,, \qquad [17]$$

where the coefficients λ_i are the eigenvalues of W_{AA} and the A_i are $(m \times m)$ matrices, which are calculated as follows [3]. Let B be the matrix whose i^{th} column vector, $\boldsymbol{b}_i = (b_{1i}, \dots, b_{mi})^T$, is the eigenvector corresponding to eigenvalue λ_i of W_{AA}. Let $\mathrm{C} = \mathrm{B}^{-1}$ be the inverse of B and $\boldsymbol{c}_i = (c_{i1}, \dots, c_{im})$ its ith row vector. Now,

$$\mathrm{A}_i = \boldsymbol{b}_i \boldsymbol{c}_i \,. \qquad [18]$$

With Eq. [18], it follows that

$$\mathrm{A}_i \mathrm{A}_j = \begin{cases} \boldsymbol{0} & if \ i \neq j \\ \mathrm{A}_i & if \ i = j \end{cases} . \qquad [19]$$

Relationships [19] imply

$$(\mathrm{W}_{AA})^k = \sum_{i \in A} (\lambda_i)^k \mathrm{A}_i \,. \qquad [20]$$

Insertion of Eq. [20] into [13] gives

([5]) Distinct eigenvalues is a sufficient (but not necessary) condition for diagonalizability.

$$
\begin{aligned}
e^{t\mathrm{W}_{AA}} &= \sum_{k=0}^{\infty} \frac{(t\mathrm{W}_{AA})^k}{k!} \\
&= \sum_{k=0}^{\infty} \sum_{i\in A} \frac{(\lambda_i t)^k}{k!} \mathrm{A}_i \\
&= \sum_{i\in A} \left(\sum_{k=0}^{\infty} \frac{(\lambda_i t)^k}{k!} \right) \mathrm{A}_i \\
&= \sum_{i\in A} e^{\lambda_i t} \mathrm{A}_i \,.
\end{aligned}
\tag{21}
$$

Insertion of Eq. [21] into Eq. [16] gives

$$f_A(t) = \boldsymbol{u}_B \mathrm{W}_{BA} \left(\sum_{i\in A} e^{\lambda_i t} \mathrm{A}_i \right) \frac{\mathrm{W}_{AB} \boldsymbol{p}_B(t)}{\boldsymbol{u}_A \mathrm{W}_{AB} \boldsymbol{p}_B(t)} \,. \tag{22}$$

Since the diagonal elements of W_{AA} are non-positive (*cf.* Eq. [2]), it follows with Gershgorin's disc theorem [2] that the real part of the eigenvalues of W_{AA} is non-positive:

$$Re(\lambda_i) \leq 0 \,. \tag{23}$$

In summary, we may state:

THEOREM 1. If W_{AA} is diagonalizable and all its eigenvalues are real, the density function of H_A, the dwell time of the random process Y in A, is given by the linear combination of decaying exponentials,

$$f_A(t) = \sum_{i\in A} c_i(t) e^{\lambda_i t} \,, \tag{24}$$

for all $i \in A$, where the $\lambda_i' s \leq 0$ are the eigenvalues of the generator submatrix W_{AA}, and

$$c_i(t) = \frac{\boldsymbol{u}_B \mathrm{W}_{BA} \mathrm{A}_i \mathrm{W}_{AB} \boldsymbol{p}_B(t)}{\boldsymbol{u}_A \mathrm{W}_{AB} \boldsymbol{p}_B(t)} \,. \tag{25}$$

Theorem 1 was proved before under the more stringent assumption of nondegenerate (*i.e.*, distinct) eigenvalues of W_{AA} [5], which implies diagonalizability (*cf.* Footnote 3).

For the sake of calculating f_A, the internal topology of B is irrelevant; what solely matters is the pattern in which probability currents reach A states from B, i.e., $\boldsymbol{r}(t)$ — *cf.* Eq. [14]. B states may therefore be combined into a single microstate with probability $p_B(t) = \boldsymbol{u}_B \boldsymbol{p}_B(t)$ as long as the probability currents between B and all $i \in A$ are preserved, i.e.,

$$w_{iB}p_B(t) = \sum_{j\in B} w_{ij}p_j(t)\,.$$

Thus W_{AB} becomes a column vector (if it wasn't a column vector already) with elements

$$w_{iB}(t) = \sum_{j\in B} w_{ij}p_j(t)/p_B(t)$$

for all $i \in A$. Since p_B is a number, it cancels out in the numerator and denominator, and Eq. [25] becomes

$$c_i(t) = \frac{\boldsymbol{u}_B \mathrm{W}_{BA}\mathrm{A}_i\mathrm{W}_{AB}(t)}{\boldsymbol{u}_A \mathrm{W}_{AB}(t)}.$$

In steady state, the time dependence may be dropped, $c_i(t) = c_i$.

COROLLARY 1. The dwell time of a Markov process in a single microstate is exponentially distributed.

Proof: Let $A = \{1\}$. Then, $\boldsymbol{u}_B\mathrm{W}_{BA} = -w_{11}$, $\mathrm{W}_{AA} = w_{11}$, $\mathrm{A}_1 = 1$, $\lambda_1 = w_{11}$, and $\boldsymbol{u}_A = 1$. With Theorem 1 it follows that

$$f_A(t) = -w_{11}e^{w_{11}t},$$

the density of the exponential distribution [3,6]. ■

II. THREE-STATE GRAPHS

A collection of nodes representing states and directed edges (arrows) that indicate possible transitions between them is called a "directed graph." In this section, we limit our discussion to processes on graphs with three nodes $S = \{1,2,3\}$, $A = \{1,2\}$ and $B = \{3\}$ (*cf.* Fig. 3A). The generator of the process is

$$\mathrm{W} = \begin{pmatrix} w_{11} & w_{12} & w_{13} \\ w_{21} & w_{22} & w_{23} \\ w_{31} & w_{32} & w_{33} \end{pmatrix}.$$

Thus

$$\mathrm{W}_{AA} = \begin{pmatrix} w_{11} & w_{12} \\ w_{21} & w_{22} \end{pmatrix}.$$

where

$$w_{11} = -w_{21} - w_{31}$$

$$w_{22} = -w_{12} - w_{32}$$

(*cf.* Eq. [2]). The eigenvalues of W_{AA}, the roots of its characteristic polynomial, are given by

$$\begin{aligned}\lambda_1 = &-\frac{1}{2}(w_{12} + w_{21} + w_{31} + w_{32}) \\ &-\frac{1}{2}\sqrt{(w_{12} + w_{21} + w_{31} + w_{32})^2 - 4(w_{12}w_{31} + w_{21}w_{32} + w_{31}w_{32})}\end{aligned} \qquad [26]$$

and

$$\lambda_2 = -\frac{1}{2}(w_{12} + w_{21} + w_{31} + w_{32}) \\ + \frac{1}{2}\sqrt{(w_{12} + w_{21} + w_{31} + w_{32})^2 - 4(w_{12}w_{31} + w_{21}w_{32} + w_{31}w_{32})} \quad [27]$$

The eigenvalues λ_1 and λ_2 are real, and distinct if at least one of the rate constants that define the eigenvalues of W_{AA} is > 0. To show this, we prove:

LEMMA 1. For $a, b, c, d \in \mathbb{R}_{\geq 0}$ and $a + b + c + d > 0$,

$$(a + b + c + d)^2 - 4(ac + bd + cd) > 0\,.$$

Proof:

$$\begin{aligned}(a + b + c + d)^2 - 4(ac + bd + cd) &= (a - c)^2 + (b - d)^2 + 2ab + 2ad + 2bc - 2cd \\ &\geq (a - c)^2 + (b - d)^2 - 2ab + 2ad + 2bc - 2cd \\ &= (c - a)^2 + (b - d)^2 + 2(c - a)(b - d) \\ &= (c - a + b - d)^2 > 0\,. \blacksquare\end{aligned}$$

With Lemma 1, $w_{12} = a$, $w_{21} = b$, $w_{31} = c$, $w_{32} = d$, and Eqs. [26] and [27], it follows that the eigenvalues of W_{AA} (λ_1 and λ_2) are real and nondegenerate. The nondegeneracy of the eigenvalues implies that W_{AA} is diagonalizable (*cf*. Footnote 3). Thus, with Theorem 1, we obtain for the density function of H_A:

$$f_A(t) = c_1 e^{\lambda_1 t} + c_2 e^{\lambda_2 t}\,. \quad [28]$$

The coefficients c_1 and c_2 are cumbersome expressions of the rate constants w_{ji}. However, the sum of the two is surprisingly simple:

$$c_1 + c_2 = \frac{w_{13}w_{31} + w_{23}w_{32}}{w_{13} + w_{23}}\,. \quad [29]$$

Unless the graph is strongly connected, some states will have zero probability in steady state. We therefore admit only strongly connected graphs. Two types of strongly connected "trees," i.e., non-cyclical graphs, stand to reason: $w_{13}, w_{31} = 0$ and $w_{12}, w_{21} = 0$ (*cf*. Fig. 3A).

THEOREM 2. For trees, $c_1, c_2 \geq 0$, i.e., f_A is monotonic.

To prove this claim, we need the following:

LEMMA 2. For $w_{31} = 0$, w_{32} and w_{21}, are bounded by λ_1 and λ_2:

$$-\lambda_2 < w_{32}, w_{21} < -\lambda_1\,.$$

Proof: To simplify notation, let $a = w_{12}$, $b = w_{21}$, and $x = w_{32}$. Since $a, b > 0$,

$$b < a + b\,,$$

which implies the following inequalities:

$$4bx < 4(a+b)x$$

$$0 < 4(a+b)x - 4bx$$

$$-2(a+b)x < 2(a+b)x - 4bx$$

$$(a+b)^2 - 2(a+b)x + x^2 < (a+b)^2 + 2(a+b)x - 4bx + x^2$$

$$(a+b-x)^2 < (a+b+x)^2 - 4bx$$

$$a+b-x < \sqrt{(a+b+x)^2 - 4bx}$$

$$a+b+x < \sqrt{(a+b+x)^2 - 4bx} + 2x$$

$$\frac{1}{2}(a+b+x) - \frac{1}{2}\sqrt{(a+b+x)^2 - 4bx} = -\lambda_2 < x\,,$$

where the equality follows from Eq. [27] and $w_{31} = 0$.

Likewise, with $(a+b-x)^2 = (x-a-b)^2$ it follows from the fifth line above that

$$x - (a+b) < \sqrt{(a+b+x)^2 - 4bx}$$

$$2x - (a+b) < x + \sqrt{(a+b+x)^2 - 4bx}$$

$$x < \frac{1}{2}(a+b+x) + \frac{1}{2}\sqrt{(a+b+x)^2 - 4bx} = -\lambda_1\,,$$

where the equality is due to Eq. [26] and $w_{31} = 0$. Thus,

$$-\lambda_2 < w_{32} < -\lambda_1.$$

Multiplication of the last inequality with $w_{21} > 0$ yields

$$-\lambda_2 w_{21} < w_{21} w_{32} < -\lambda_1 w_{21}$$

and with $\lambda_1 \lambda_2 = w_{21} w_{32}$ it follows that

$$-\lambda_2 < w_{21} < -\lambda_1\,. \blacksquare$$

Proof of Theorem 2: For $w_{12}, w_{21} = 0$,

$$\boldsymbol{r}(t) = \frac{1}{w_{13} + w_{23}} \begin{pmatrix} w_{13} \\ w_{23} \end{pmatrix},$$

$$A_1 = \begin{pmatrix} 1 & 0 \\ 0 & 0 \end{pmatrix},$$

$$A_2 = \begin{pmatrix} 0 & 0 \\ 0 & 1 \end{pmatrix},$$

$\boldsymbol{u}_B = 1$, $\mathrm{W}_{BA} = (w_{31}, w_{32})$, $\lambda_1 = -w_{31}$ and $\lambda_2 = -w_{32}$ (*cf.* Eq.'s, [26] and [27])

$$c_1 = \boldsymbol{u}_B \mathrm{W}_{BA} \mathrm{A}_1 \boldsymbol{r} = -r_1 \lambda_1 > 0\,,$$

$$c_2 = \boldsymbol{u}_B \mathrm{W}_{BA} \mathrm{A}_2 \boldsymbol{r} = -r_2 \lambda_2 > 0\,.$$

The inequality sign follows with Eq.'s, [26] and [27]. This proves Theorem 2 for $w_{12}, w_{21} = 0$.

For $w_{13}, w_{31} = 0$,

$$\boldsymbol{r}(t) = \begin{pmatrix} 0 \\ 1 \end{pmatrix},$$

$$A_1 = -\frac{1}{\lambda_2 - \lambda_1}\begin{pmatrix} \lambda_2 + w_{21} & w_{12} \\ w_{21} & \lambda_1 + w_{21} \end{pmatrix},$$

$$A_2 = \frac{1}{\lambda_2 - \lambda_1}\begin{pmatrix} -\lambda_1 - w_{21} & w_{12} \\ w_{21} & \lambda_2 + w_{21} \end{pmatrix},$$

$\boldsymbol{u}_B = 1$ and $\mathrm{W}_{BA} = (0, w_{32})$. Thus, with $\lambda_1\lambda_2 = w_{32}w_{21}$, it follows that

$$c_1 = \boldsymbol{u}_B \mathrm{W}_{BA} \mathrm{A}_1 \boldsymbol{r} = -\frac{w_{32}(\lambda_1 + w_{21})}{\lambda_2 - \lambda_1} = -\frac{w_{32} + \lambda_2}{\lambda_2 - \lambda_1}\lambda_1$$

and

$$c_2 = \boldsymbol{u}_B \mathrm{W}_{BA} \mathrm{A}_2 \boldsymbol{r} = \frac{w_{32}(\lambda_2 + w_{21})}{\lambda_2 - \lambda_1} = \frac{w_{32} + \lambda_1}{\lambda_2 - \lambda_1}\lambda_2 \,.$$

With Lemma 2 it follows that $w_{32} + \lambda_2 > 0$ and $w_{32} + \lambda_1 < 0$. Since $\lambda_2 - \lambda_1 > 0$ and $\lambda_1 < 0$, we obtain $c_1, c_2 \geq 0$. This proves Theorem 2 for $w_{13}, w_{31} = 0$. ■

THEOREM 3. Only cyclical graphs with unidirectional entry into and exit out of A allow for $c_1 + c_2 = 0$.

Proof: With Eq. [29] it is seen that $c_1 + c_2 = 0$ if and only if $w_{13}w_{31} + w_{23}w_{32} = 0$. Only two strongly connected graphs fulfill this requirement: first, the graph with $w_{13}, w_{32} = 0$ and $w_{31}, w_{23} > 0$; second, the graph with $w_{31}, w_{23} = 0$ and $w_{13}, w_{32} > 0$. In either case, entry into and exit out of A are unidirectional (*cf*. Fig. 3A). In contrast, $w_{13}, w_{31}, w_{32} = 0$ satisfies $w_{13}w_{31} + w_{23}w_{32} = 0$, but the graph is neither strongly connected nor cyclical. The same is true for all remaining possibilities that satisfy $w_{13}w_{31} + w_{23}w_{32} = 0$. ■

By integration of Eq. [28], we obtain the distribution for the dwell time in A:

$$F_A(t) = 1 + \frac{c_1}{\lambda_1}e^{\lambda_1 t} + \frac{c_2}{\lambda_2}e^{\lambda_2 t} \,.$$

With $c_1 + c_2 = 0$ and $F_A(0) = 0$, it follows that

$$c_1 = \lambda_1\lambda_2/(\lambda_1 - \lambda_2)$$

and

$$c_2 = \lambda_1\lambda_2/(\lambda_2 - \lambda_1) \,.$$

Since the eigenvalues of W_{AA} only depend on rate constants for transitions within and out of A, the dwell time density for A is independent of the rate constants for entry into A (w_{13} or w_{23}). (The transformation of a maximally peaked into a non-peaked dwell

time density, as in the case of the activator mutant, cannot be explained, therefore, by a decrease in the rate constant for entry into A.)

III. TU'S THEOREM

We prove that the monotonicity of dwell time densities is a Hopfield barrier, in other words:

THEOREM 4. If Y is reversible — i.e., the detailed balance conditions for equilibrium are fulfilled — f_A, the probability density for the dwell time of Y in mesostate A, is the sum of decaying exponentials with non-negative weights c_i [7,8].

Proof: That f_A is the weighted sum of decaying exponentials if W_{AA} is diagonalizable and its eigenvalues are real was proved above (Theorem 1). Here, we show that the generator of a reversible process is similar (conjugated) to a symmetric matrix, which implies all we need: real eigenvalues, diagonalizability, and non-negative exponential weights. (The latter, then, implies the monotonicity of f_A.)

The following proof follows Skinner and Dunkel (2021)[8]; the only difference, beside nomenclature, is that our probability mass function is a column and not row vector — corresponding matrices are therefore related to each other by transposition — and that we lay out the argument in more detail, hoping to increase accessibility without obfuscating the proof's central idea.

First, we recast Eq. [16] on the assumption that Y is stationary, i.e.,

$$\mathrm{W}\boldsymbol{p} = \begin{pmatrix} \mathrm{W}_{AA} & \mathrm{W}_{AB} \\ \mathrm{W}_{BA} & \mathrm{W}_{BB} \end{pmatrix} \begin{pmatrix} \boldsymbol{p}_A \\ \boldsymbol{p}_B \end{pmatrix} = \begin{pmatrix} \mathbf{0} \\ \mathbf{0} \end{pmatrix},$$

where $\boldsymbol{p} = (\boldsymbol{p}_A, \boldsymbol{p}_B)^T$ is the stationary distribution of Y. The previous equation implies that

$$\mathrm{W}_{AA}\boldsymbol{p}_A + \mathrm{W}_{AB}\boldsymbol{p}_B = \mathbf{0}$$

and thus

$$-\mathrm{W}_{AA}\boldsymbol{p}_A = \mathrm{W}_{AB}\boldsymbol{p}_B \,. \qquad [30]$$

Furthermore, by virtue of Eq. [2],

$$(\boldsymbol{u}_A, \boldsymbol{u}_B) \begin{pmatrix} \mathrm{W}_{AA} & \mathrm{W}_{AB} \\ \mathrm{W}_{BA} & \mathrm{W}_{BB} \end{pmatrix} = (\mathbf{0}, \mathbf{0})\,;$$

whence

$$-\boldsymbol{u}_A\mathrm{W}_{AA} = \boldsymbol{u}_B\mathrm{W}_{BA} \,. \qquad [31]$$

Insertion of Eqs. [30] and [31] into Eq. [16] yields

$$f_A(t) = -\frac{\boldsymbol{u}_A\mathrm{W}_{AA}e^{t\mathrm{W}_{AA}}\mathrm{W}_{AA}\boldsymbol{p}_A}{\boldsymbol{u}_A\mathrm{W}_{AA}\boldsymbol{p}_A} \,.$$

Since W_{AA} and $e^{t\mathrm{W}_{AA}}$ commute — this may easily be seen by writing out the Taylor expansion of the latter (*cf.* Eq. [13]) — we may write

$$f_A(t) = \frac{\boldsymbol{u}_A e^{t\mathrm{W}_{AA}} \mathrm{W}_{AA}^2 \boldsymbol{p}_A}{v_{AB}} \ , \qquad [32]$$

where $v_{AB} = -\boldsymbol{u}_A \mathrm{W}_{AA} \boldsymbol{p}_A = \boldsymbol{u}_A \mathrm{W}_{AB} \boldsymbol{p}_B$; this is Lemma 1 in Skinner and Dunkel, where it is stated without proof [8].

We now assume that the process is not only stationary but reversible — i.e., the detailed balance conditions are fulfilled[9]:

$$w_{ij}\pi_j = w_{ji}\pi_i \qquad [33]$$

for all $i, j \in A$, where $\boldsymbol{\pi} = \boldsymbol{p}$ is the equilibrium distribution of Y. We define a new matrix,

$$D = \Lambda^{-1} \mathrm{W}_{AA} \Lambda \, , \qquad [34]$$

where Λ is the diagonal matrix with (diagonal) elements $\pi_1^{1/2}, \dots, \pi_m^{1/2}$ (from the top to bottom row). The diagonal elements of its inverse Λ^{-1}, therefore, are $\pi_1^{-1/2}, \dots, \pi_m^{-1/2}$.

Two matrices that are related to one another by virtue of an invertible matrix in the form of Eq. [34] are said to be "similar" or "conjugated." Similar matrices have the same characteristic polynomial and thus identical eigenvalues (although not identical eigenvectors):

$$\begin{aligned} det(\lambda I - D) &= det(\lambda \Lambda^{-1} I \Lambda - \Lambda^{-1} \mathrm{W}_{AA} \Lambda) \\ &= det(\Lambda^{-1}(\lambda I - \mathrm{W}_{AA})\Lambda) \\ &= det(\Lambda^{-1}\Lambda) det(\lambda I - \mathrm{W}_{AA}) \\ &= det(\lambda I - \mathrm{W}_{AA}) \, , \end{aligned}$$

where $det(X)$ is the determinant of matrix X and I is the identity matrix. Importantly, $D = \{d_{ij}\}$ is symmetric, since

$$d_{ij} = \pi_i^{-1/2} w_{ij} \pi_j^{1/2} = \pi_j^{-1/2} w_{ji} \pi_i^{1/2} = d_{ji} \, ,$$

where the second equality sign is due to the detailed balance condition, Eq. [33].

Symmetry has important implications. First, the eigenvalues of a symmetric real matrix are real: Let λ be a potentially complex eigenvalue of D with corresponding eigenvector $\boldsymbol{z}$, i.e., $D\boldsymbol{z} = \lambda \boldsymbol{z}$. With $D = D^T = \bar{D}$, where the overbar indicates the complex conjugate, follows

$$\lambda \bar{\boldsymbol{z}}^T \boldsymbol{z} = \bar{\boldsymbol{z}}^T \lambda \boldsymbol{z} = \bar{\boldsymbol{z}}^T D \boldsymbol{z} = (D^T \bar{\boldsymbol{z}})^T \boldsymbol{z} = (\bar{D}\bar{\boldsymbol{z}})^T \boldsymbol{z} = \left(\bar{\lambda}\bar{\boldsymbol{z}}\right)^T \boldsymbol{z} = \bar{\lambda} \bar{\boldsymbol{z}}^T \boldsymbol{z} \, .$$

Thus, $\lambda = \bar{\lambda}$, since $\bar{\boldsymbol{z}}^T \boldsymbol{z} \neq 0$ *i.e.*, λ is real. Second, a symmetric $(m \times m)$ matrix, D, has m orthonormal eigenvectors: Let $\boldsymbol{z}_1$ and $\boldsymbol{z}_2$ be two eigenvectors of D and λ_1 and λ_2 their corresponding eigenvalues, *i.e.*, $D\boldsymbol{z}_1 = \lambda_1 \boldsymbol{z}_1$ and $D\boldsymbol{z}_2 = \lambda_2 \boldsymbol{z}_2$. Then,

$$\lambda_1 \boldsymbol{z}_2^T \boldsymbol{z}_1 = \boldsymbol{z}_2^T D \boldsymbol{z}_1 = \boldsymbol{z}_2^T D^T \boldsymbol{z}_1 = (\boldsymbol{z}_1^T D \boldsymbol{z}_2)^T = (\lambda_2 \boldsymbol{z}_1^T \boldsymbol{z}_2)^T = \lambda_2 \boldsymbol{z}_2^T \boldsymbol{z}_1 .$$

It follows that either $\boldsymbol{z}_2^T \boldsymbol{z}_1 = 0$, i.e., $\boldsymbol{z}_1$ and $\boldsymbol{z}_2$ are orthogonal, or $\lambda_1 = \lambda_2$, i.e., $\boldsymbol{z}_1, \boldsymbol{z}_2$ belong to the same eigenspace. With Gram-Schmidt, an orthogonal basis may be constructed for any linear space. It thus is possible to find an orthonormal basis of eigenvectors of D.

If Z is the matrix whose column vectors $\boldsymbol{z}_i$ are orthonormal eigenvectors of D, then $Z^T Z = I$. Thus, the inverse of Z is equal to its transpose,

$$Z^{-1} = Z^T . \qquad [35]$$

Since orthonormal vectors are linearly independent, D is diagonalizable. By virtue of the spectral decomposition theorem (*cf.* Eq. [17] to [19]), we may write D, therefore, as a linear combination of square matrices,

$$D = \sum_{i \in A} \lambda_i E_i , \qquad [36]$$

where the λ_i are the (real) eigenvalues of W_{AA} (and thus of D) and E_i is the square matrix

$$E_i = \boldsymbol{z}_i (\boldsymbol{z}_i)^T = \begin{pmatrix} z_{1i} z_{1i} & \cdots & z_{1i} z_{mi} \\ \vdots & \ddots & \vdots \\ z_{mi} z_{1i} & \cdots & z_{mi} z_{mi} \end{pmatrix},$$

where the first equality sign is due to Eqs. [18] and [35].

Eq. [34] implies that $\mathrm{W}_{AA} = \Lambda D \Lambda^{-1}$. Insertion of this latter expression into Eq. [32] gives

$$\begin{aligned} f_A(t) &= \frac{\boldsymbol{u}_A e^{t \Lambda D \Lambda^{-1}} (\Lambda D \Lambda^{-1})^2 \boldsymbol{\pi}_A}{v_{AB}} \\ &= \frac{\boldsymbol{u}_A \Lambda e^{tD} \Lambda^{-1} (\Lambda D \Lambda^{-1})^2 \boldsymbol{\pi}_A}{v_{AB}} \\ &= \frac{\boldsymbol{u}_A \Lambda e^{tD} D^2 \Lambda^{-1} \boldsymbol{\pi}_A}{v_{AB}} . \end{aligned} \qquad [37]$$

For the second equality sign we used $(\Lambda D \Lambda^{-1})^k = \Lambda D^k \Lambda^{-1}$ for all $k \in \mathbb{N}$. Now,

$$D^2 = \sum_{i \in A} \lambda_i^2 E_i \qquad [38]$$

(*cf.* Eq. [19]), and

$$e^{tD} = \sum_{i \in A} e^{\lambda_i t} E_i \qquad [39]$$

(*cf.* Eq.'s [13] to [21]). With Eq. [19], we obtain from the previous two equations

$$e^{tD} D^2 = \sum_{i \in A} \lambda_i^2 e^{\lambda_i t} E_i .$$

Insertion of the last equation into Eq. [37] gives

$$f_A(t) = \frac{1}{v_{AB}} \boldsymbol{u}_A \Lambda \left(\sum_{i \in A} \lambda_i^2 e^{\lambda_i t} E_i \right) \Lambda^{-1} \boldsymbol{\pi}_A$$

$$= \frac{1}{v_{AB}} \sum_{i \in A} \lambda_i^2 e^{\lambda_i t} \boldsymbol{u}_A \Lambda E_i \Lambda^{-1} \boldsymbol{\pi}_A \,.$$

With

$$\Lambda E_i \Lambda^{-1} = \begin{pmatrix} \pi_1^{1/2} z_{1i} z_{1i} \pi_1^{-1/2} & \cdots & \pi_1^{1/2} z_{1i} z_{mi} \pi_m^{-1/2} \\ \vdots & \ddots & \vdots \\ \pi_m^{1/2} z_{mi} z_{1i} \pi_1^{-1/2} & \cdots & \pi_m^{1/2} z_{mi} z_{mi} \pi_m^{-1/2} \end{pmatrix}$$

follows

$$\Lambda E_i \Lambda^{-1} \boldsymbol{\pi}_A = \begin{pmatrix} \sum_{k \in A} \pi_1^{1/2} z_{1i} z_{ki} \pi_k^{1/2} \\ \vdots \\ \sum_{k \in A} \pi_s^{1/2} z_{si} z_{ki} \, \pi_k^{1/2} \end{pmatrix}.$$

Multiplication from the left with $\boldsymbol{u}_A$ gives

$$\boldsymbol{u}_A \Lambda E_i \Lambda^{-1} \boldsymbol{\pi}_A = \sum_{j,k \in A} \pi_j^{1/2} z_{ji} z_{ki} \, \pi_k^{1/2}$$

$$= \left(\sum_{j \in A} \pi_j^{1/2} z_{ji} \right) \left(\sum_{k \in A} \pi_k^{1/2} z_{ki} \right)$$

$$= \left(\left(\boldsymbol{\pi}_A^{1/2} \right)^T \boldsymbol{z}_i \right)^2,$$

where $\boldsymbol{\pi}_A^{1/2} = \left(\pi_1^{1/2}, \ldots, \pi_m^{1/2} \right)$. By inserting this last equation into Eq. [37], we obtain

$$f_A(t) = \sum_{i \in A} \frac{\lambda_i^2}{v_{AB}} \left(\left(\boldsymbol{\pi}_A^{1/2} \right)^T \boldsymbol{z}_i \right)^2 e^{\lambda_i t} \,,$$

With $v_{AB} \geq 0$, since all the coefficients of W_{AB} are non-negative, $\left(\left(\boldsymbol{\pi}_A^{1/2} \right)^T \boldsymbol{v}_i \right)^2 \geq 0$, and $\lambda_i^2 \geq 0$ for all $i \in A$ follows the claim. ■

IV. ENTROPY PRODUCTION OF COARSE-GRAINED PROCESSES

THEOREM 5. The entropy production of any coarse-grained process provides a lower bound to the true entropy production [8,10].

Proof: We shall need the following "log-sum inequality:"

$$\sum_j a_j ln\left(\frac{a_j}{b_j}\right) \geq \left(\sum_j a_j\right) ln\left(\frac{\sum_j a_j}{\sum_j b_j}\right),$$

for all $a_j, b_j > 0$, $j = 1, \dots, n$.[11]

Let i, j, k be microstates where i, j are indistinguishable to the observer, who combines them to mesostate $A = \{i, j\}$. Let $v_{jk}(t)$ be the current of probability mass from microstate $k \notin A$ to microstate j at time t; $v_{jk}(t)$, then, is the corresponding countercurrent, from k to j. In the following, we shall omit the time-dependence for simplicity's sake. The probability currents between A and microstate k are

$$v_{kA} = v_{kj} + v_{ki}\ ,$$
$$v_{Ak} = v_{jk} + v_{ik}\ .$$

The true entropy production, σ, is the sum of the entropy production along all edges between microstates [12],

$$\sigma = \frac{k_B}{2} \sum_{j,k \in S} (v_{jk} - v_{kj}) ln\left(\frac{v_{jk}}{v_{kj}}\right).$$

If none or only one microstate of A is connected to microstate k, the coarse-graining (the combination of microstates i and j to a single state A, simply eliminates the edge between $i, j \in A$; this can only reduce entropy production but does not change the entropy production along the edge that connects A to k. If both $i, j \in A$ are connected to $k \notin A$, the true entropy production contains the partial sum

$$\begin{aligned}
&(v_{kj} - v_{jk}) ln\left(\frac{v_{kj}}{v_{jk}}\right) + (v_{ki} - v_{ik}) ln\left(\frac{v_{ki}}{v_{ik}}\right) \\
&= v_{kj} ln\left(\frac{v_{kj}}{v_{jk}}\right) + v_{ki} ln\left(\frac{v_{ki}}{v_{ik}}\right) + v_{jk} ln\left(\frac{v_{jk}}{v_{kj}}\right) + v_{ik} ln\left(\frac{v_{ik}}{v_{ki}}\right) \\
&\geq (v_{kj} + v_{ki}) ln\left(\frac{v_{kj} + v_{ki}}{v_{jk} + v_{ik}}\right) + (v_{jk} + v_{ik}) ln\left(\frac{v_{jk} + v_{ik}}{v_{kj} + v_{ki}}\right) \\
&= (v_{kj} + v_{ki}) ln\left(\frac{v_{kj} + v_{k_1 i}}{v_{jk} + v_{ik}}\right) - (v_{jk} + v_{ik}) ln\left(\frac{v_{kj} + v_{ki}}{v_{jk} + v_{ik}}\right) \\
&= \left((v_{kj} + v_{ki}) - (v_{jk} + v_{ik})\right) ln\left(\frac{v_{kj} + v_{ki}}{v_{jk} + v_{ik}}\right) \\
&= (v_{kA} - v_{Ak}) ln\left(\frac{v_{kA}}{v_{Ak}}\right),
\end{aligned}$$

where the inequality is obtained by applying the log-sum inequality to the first and second pair of summands of the sum above the inequality sign. The final term (last line) is the entropy production of the coarse-grained process (microstates i and j combined to

A) due to probability currents between A and k. This calculation may be repeated for all $k \notin A$. Any level of coarse-graining may be reached by successively combining microstates to mesostates, which may decrease but not increase entropy production. Thus, the entropy production of any coarse-grained version of the original process provides a lower bound on the true entropy production. ■

We note that the above proof does not invoke the Markov assumption; the proof does not require that probability currents are linear functions of microstate probabilities (*cf*. Eq. [1]). It does depend, however, on the validity of stochastic thermodynamics, specifically the postulate that entropy production is a logarithmic function of the ratios of forward and corresponding reverse probability currents [12], the proof's starting point.

EXTENDED DATA FIGURES

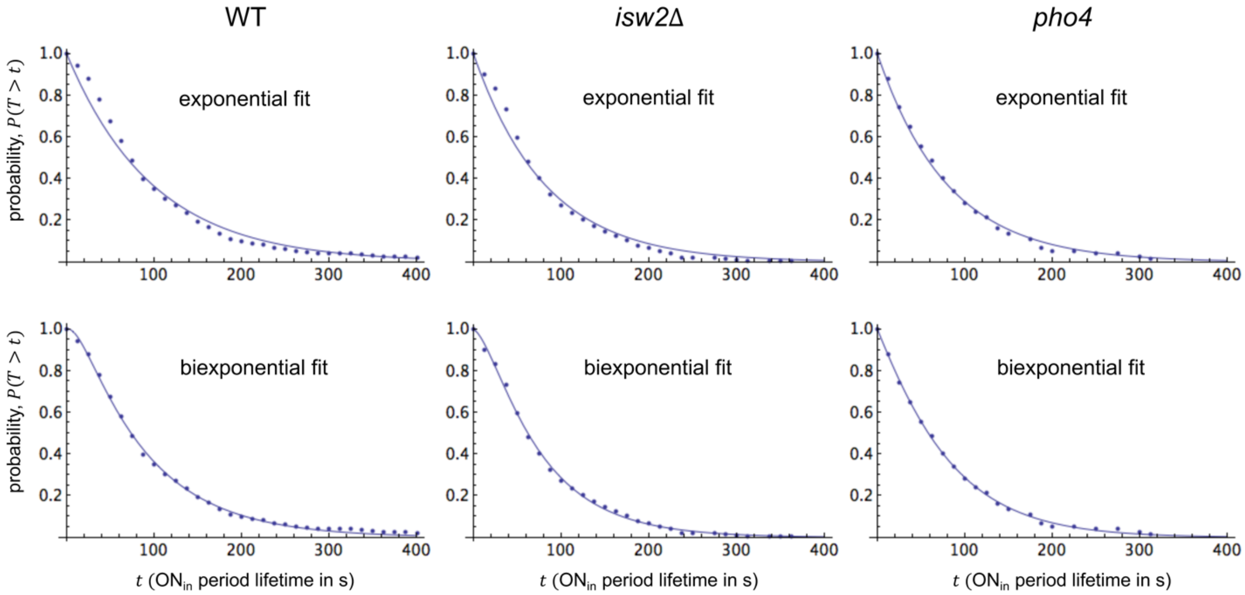

Extended Data, Fig. 1. Exponential and biexponential fits to survival curves of internal ON periods. Measurements are indicated by blue dots, fitted model graphs by continuous blue line; top row: one-exponential fits, $e^{-\lambda t}$; bottom row: two-exponential fits, $-ae^{-\lambda_1 t} + (1 + a)e^{-\lambda_2 t}$. First column: wild type; second column: *isw2*Δ; third column: *pho4* mutant. For wild type we analyzed 218 sample paths (i.e., cells over a time window of 20 minutes); for isw2Δ, 291 sample paths, and for *pho4*Δ[75-90], 158 sample paths.

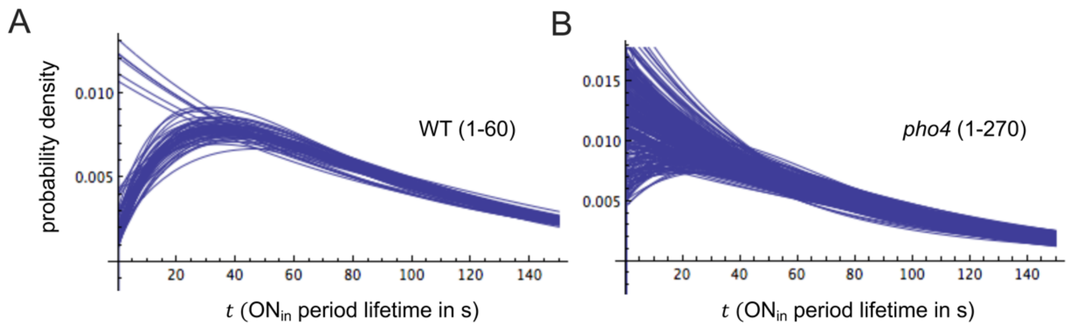

Extended Data, Fig. 2. Nearly all ON bootstrap-densities were manifestly peaked for wild type but not activator mutant. Density functions (300), generated by bootstrapping of internal ON-period lengths, were ordered according to decreasing density at $t = 0.01$ seconds. (Density were evaluated close to zero, $t = 0.01$ s, and not at zero, because some bootstrap density functions were virtually discontinuous at $t = 0$ with a large negative density at $t = 0$, due to a large λ_1, but positive density for $t > \varepsilon$, where ε is a number close to zero.) (a) The first 60 of 300 bootstrap densities for wild type. (b) The first 270 of 300 bootstrap densities for the *pho4* mutant.

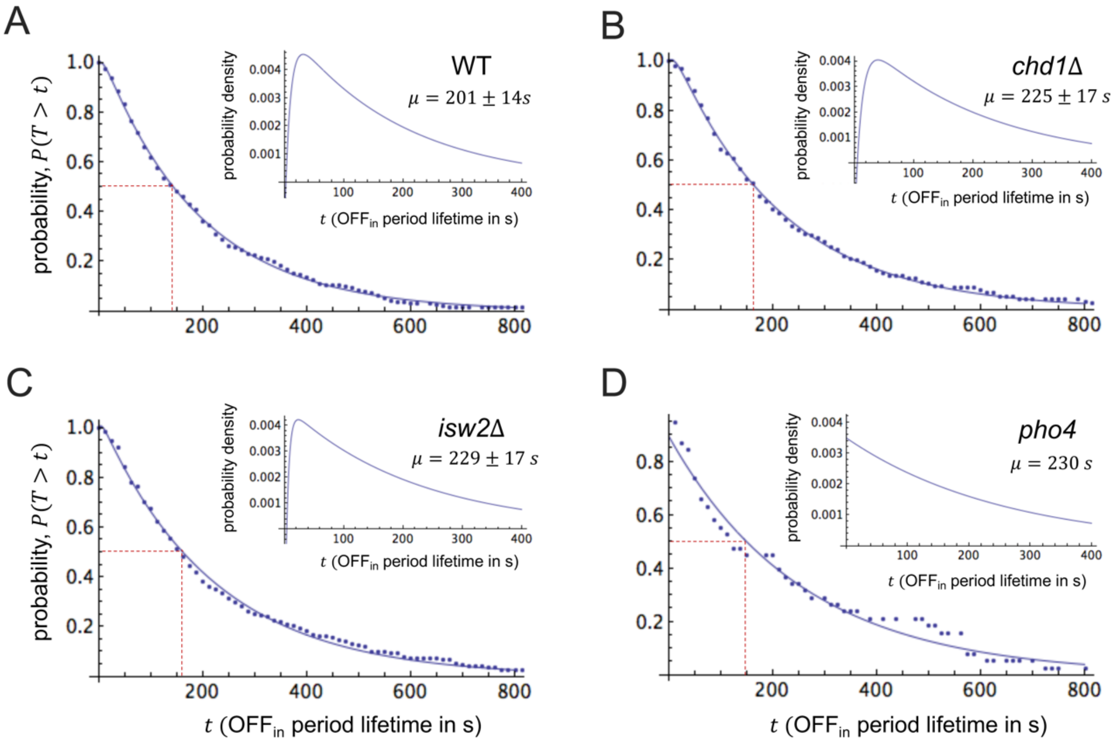

Extended Data, Fig. 3. Internal OFF-period densities were peaked, except the activator mutant. Survival curves and corresponding densities of internal OFF-period lengths for wild type (a), *chd1*Δ (b), *isw2*Δ (c), and *pho4* (d). Measurements are represented by blue dots, two-exponential fits by blue curves. Many fewer internal OFF periods were observed for the activator mutant, *pho4*, which explains the less smooth appearance of

the measurements. For wild type we analyzed 218 sample paths (cells); for *chd1*Δ, 205 cells; for isw2Δ, 291 cells, and for *pho4*Δ[75-90], 158 cells.

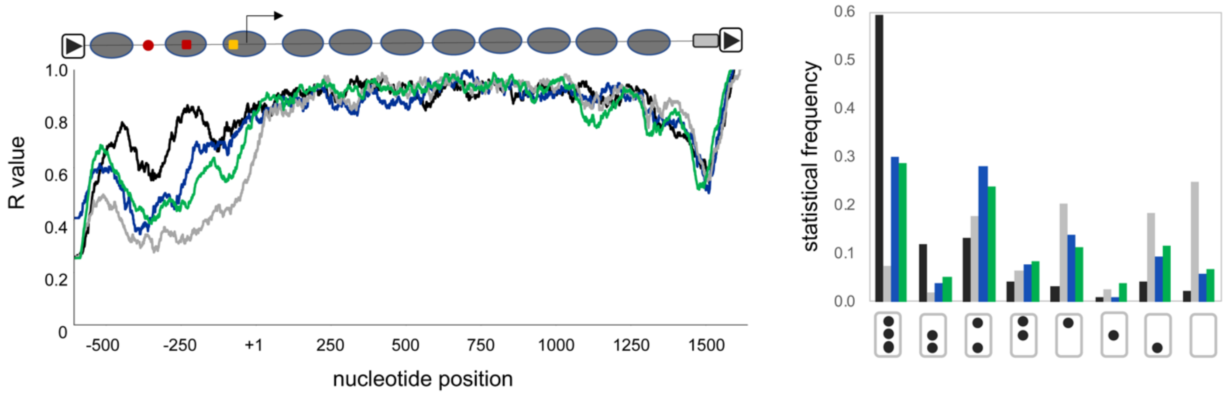

Extended Data, Fig. 4. Activator and chromatin remodeler mutations increase promoter nucleosome occupancy. Promoter nucleosome configurations from EM analysis of psoralen-crosslinked *PHO5* molecules. Left panel: Plot of R-value across the *PHO5* gene for *PHO80* (black, *PHO5* is repressed), *pho80*Δ (gray, *PHO5* is fully active), *pho4*Δ[85-99] *pho80*Δ (blue), and *chd1*Δ *pho80*Δ (green). The R-value indicates the relative frequency of finding a nucleotide position single-stranded after psoralen-crosslinking of isolated *PHO5* molecules and DNA denaturation; the R value, thus, may be seen as a measure of nucleosome occupancy, for psoralen crosslinks linker DNA, and not core particle DNA[14]. A diagram of the *PHO5* gene is shown on top. Right panel: Statistical frequencies of eight distinct nucleosome configurations for all four strains. The promoter is represented by a rectangle, occupied nucleosome positions by black dots. The 5'-terminal nucleosome is at the bottom, the 3'-terminal nucleosome at the top. Note that the *PHO5* promoter nucleosome distributions for *chd1*Δ and *pho4*Δ[85-99] (analyzed earlier[15]) were closely similar. In contrast, *PHO5* expression remained nearly five-fold stronger in the *chd1*Δ strain than in *pho4*Δ[85-99] cells [13], suggesting that Pho4 controlled *PHO5* expression not only *via* the recruitment of chromatin remodelers, in agreement with our finding that Pho4 controlled both the frequency and duration of burst runs. EM analyses were performed as detailed earlier[14,15].

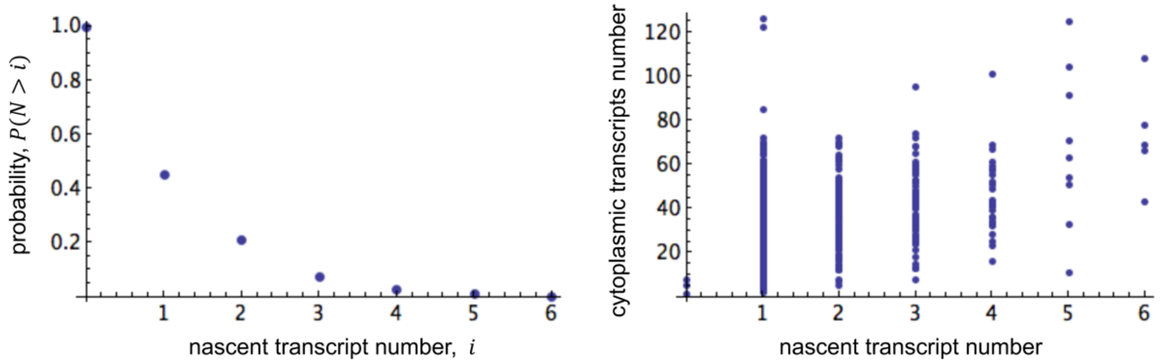

Extended Data, Fig. 5. ON periods correspond to bursts of transcription. Single molecule FISH (fluorescence in situ hybridization) analysis of *PHO5* expression. Left panel: Probability, $P(N > i)$, that the number of nascent transcripts N, given that the gene is transcriptionally active, exceeds $i = 0, 1, \cdots$. The number of nascent transcripts was determined by dividing the fluorescence intensity of the nuclear punctum by the average intensity of cytoplasmic puncta, which we assumed represented single transcripts. The closest integer to this ratio is the number of nascent transcripts. A large fraction, 45%, of *PHO5* genes bore more than one nascent transcript, suggesting that the *PHO5* gene is transcriptionally active at least 45% of the time. In contrast, MFM suggested that *PHO5* was transcriptionally active only 22% of the time. Notably, this latter percentage almost exactly equals $P(N > 2) = 0.21$ obtained from FISH, suggesting that at least 3 nascent transcripts were required to observe *PHO5* transcription by MFM. Right panel: The number of nascent transcripts (abscissa) is plotted against the number of cytoplasmic transcripts (ordinate) for 546 cells (each one of which is represented by a single blue dot). The poor correlation between nascent transcript number and cytoplasmic transcript number points to a markedly shorter lifetime of the nascent transcript compared to the cytoplasmic transcript, as expected: the half-live of the *PHO5* transcript is 10 minutes [15], 20 times longer than the lifetime of the nascent transcript, assuming a transcription rate of 50 nucleotides per seconds.

CITATIONS

1. Cinlar, E. *Introduction to Stochastic Processes*. (Dover Publications, Mineola, New York, 2013).

2. Mirzaev, I. & Gunawardena, J. Laplacian Dynamics on General Graphs. *Bulletin of Mathematical Biology* **75**, 2118–2149 (2013).

3. Cinlar, E. *Introduction to Stochastic Processes*. (Dover Publications, Mineola, New York, 2013).

4. Boeger, H. Kinetic Proofreading. *Annu Rev Biochem* **91**, 423–447 (2022).

5. D . Colquhoun and A . G . Hawkes. On the Stochastic Properties of Single Ion Channels. *Proceedings of the Royal Society of London . Series B, Biological Sciences.* **211**, 205–235 (1981).

6. Grimmet, G. & Stirzaker, D. *Probability and Random Processes*. (Oxford university press, 2001).

7. Tu, Y. The nonequilibrium mechanism for ultrasensitivity in a biological switch: Sensing by Maxwell's demons. *Proceedings of the National Academy of Sciences of the United States of America* **105**, 11737–11741 (2008).

8. Skinner, D. J. & Dunkel, J., J. Estimating Entropy Production from Waiting Time Distributions. *PHYSICAL REVIEW LETTERS* **127**, (2021).

9. Kelly, F. P. *Reversibility and Stochastic Networks*. (John Wiley and Sons Ltd, 1979).

10. Seifert, U. From Stochastic Thermodynamics to Thermodynamic Inference. *Annu. Rev. Condens. Matter Phys.* **10**, 171–192 (2019).

11. Thomas, J. A., C., T. M. *Elements of Information Theory*. (John Wiley & Sons, Inc., Publications, 2006).

12. Van den Broeck, C. Stochastic thermodynamics: A brief introduction. in *Physics of Complex Colloids* 155–193 (IOS Press, 2013). doi:10.3254/978-1-61499-278-3-155.

13. Mao, C. *et al.* Quantitative analysis of the transcription control mechanism. *Molecular Systems Biology* **6**, 1–12 (2010).

14. Brown, C. R. *et al.* Chromatin structure analysis of single gene molecules by psoralen cross-linking and electron microscopy. *Methods in Molecular Biology* **1228**, 93–121 (2015).

15. Brown, C. R., Mao, C., Falkovskaia, E., Jurica, M. S. & Boeger, H. Linking stochastic fluctuations in chromatin structure and gene expression. *PLoS Biol* **11**, e1001621 (2013).
